# Substrate-Dependent Sensitivity of SIRT1 to Nicotinamide Inhibition

**DOI:** 10.3390/biom11020312

**Published:** 2021-02-18

**Authors:** Stacia Rymarchyk, Wenjia Kang, Yana Cen

**Affiliations:** 1 Department of Pharmaceutical Sciences, Albany College of Pharmacy and Health Sciences, Colchester, VT 05446, USA; srymarchyk@yahoo.com; 2 Department of Medicinal Chemistry, Virginia Commonwealth University, Richmond, VA 23219, USA; kangw2@vcu.edu; 3 Institute for Structural Biology, Drug Discovery and Development, Virginia Commonwealth University, Richmond, VA 23219, USA

**Keywords:** SIRT1, epigenetics, deacetylation, NAM inhibition, base exchange, solvent isotope effect

## Abstract

SIRT1 is the most extensively studied human sirtuin with a broad spectrum of endogenous targets. It has been implicated in the regulation of a myriad of cellular events, such as gene transcription, mitochondria biogenesis, insulin secretion as well as glucose and lipid metabolism. From a mechanistic perspective, nicotinamide (NAM), a byproduct of a sirtuin-catalyzed reaction, reverses a reaction intermediate to regenerate NAD^+^ through “base exchange”, leading to the inhibition of the forward deacetylation. NAM has been suggested as a universal sirtuin negative regulator. Sirtuins have evolved different strategies in response to NAM regulation. Here, we report the detailed kinetic analysis of SIRT1-catalyzed reactions using endogenous substrate-based synthetic peptides. A novel substrate-dependent sensitivity of SIRT1 to NAM inhibition was observed. Additionally, SIRT1 demonstrated pH-dependent deacetylation with normal solvent isotope effects (SIEs), consistent with proton transfer in the rate-limiting step. Base exchange, in contrast, was insensitive to pH changes with no apparent SIEs, indicative of lack of proton transfer in the rate-limiting step. Consequently, NAM inhibition was attenuated at a high pH in proteated buffers. Our study provides new evidence for “activation by de-repression” as an effective sirtuin activation strategy.

## 1. Introduction

Human sirtuins are a group of NAD^+^-dependent protein lysine deacylases with distinct cellular compartmentalization and biological functions [1,2]. SIRT1, SIRT6 and SIRT7 are primarily localized in the nucleus, SIRT2 is mainly a cytosolic enzyme and SIRT3, SIRT4 and SIRT5 reside in the mitochondria [3,4]. By removing the acyl modifications from various cellular targets, sirtuins regulate diverse biological processes including metabolism [5,6], gene transcription [7], genome integrity [8] and cell cycle progression [9]. Interestingly, several human sirtuins have demonstrated increased gene expression or protein abundance in response to calorie restriction (CR) [10,11,12]. Sirtuin activities are thought to mediate the beneficial effects of CR in lifespan extension [13,14]. The last couple of decades have witnessed tremendous progress in understanding the function, disease relevance as well as developing small molecule regulators of human sirtuins [15,16,17,18].

The current study focuses on one particular human sirtuin isoform, SIRT1. Originally designated as a class III “histone deacetylase” (HDAC) [19,20], SIRT1 demonstrates good substrate tolerance by targeting a broad spectrum of cellular protein substrates, such as histones [21,22], transcription factors [23,24], tumor suppressors [25,26,27] and metabolic enzymes [28]. Through deacetylating the above-mentioned endogenous targets, SIRT1 exerts its far-reaching functions in insulin secretion [29], mitochondria biogenesis [30], DNA damage repair [31] and glucose metabolism [32]. Recent studies suggested that, in addition to the deacetylase activity, SIRT1 also removes acyl groups from lysine residues [33,34], although the physiological relevance of these novel posttranslational modifications are yet to be established. 

**Scheme 1:**
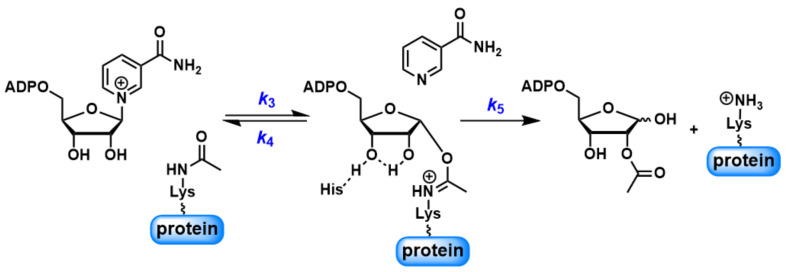
Step-by-step mechanism of sirtuin-catalyzed NAD^+^-dependent protein deacetylation.

Unlike the zinc-dependent HDACs, sirtuins employ a unique reaction mechanism for the seemingly simple deacetylation in which NAD^+^ serves as the co-substrate (Scheme 1) [35]. Cleavage of the *N*-glycosidic bond leads to the removal of nicotinamide (NAM) and the formation of an imidate intermediate. Subsequently, the imidate can be captured by the 2′-hydroxyl group of the ribose ring to generate a bicyclic intermediate. Ultimately, the collapse of the bicyclic intermediate gives rise to the deacetylated lysine product as well as a novel metabolite *O*-acetyl-ADPribose (AADPR). Detailed kinetic analysis indicated that NAM can intercept the imidate intermediate to regenerate NAD^+^ through a “base exchange” process [36]. The unusual reaction mechanism allows sirtuin activity to be tightly regulated by both NAD^+^ and NAM [37]. There is ample literature precedentsuggesting that sirtuins have evolved to respond to intracellular NAD^+^ availability [38]. For example, inhibition of the critical NAD^+^ biosynthetic enzyme, nicotinamide phosphoribosyltransferase (NAMPT), by small molecule FK866 significantly reduced the cellular NAD^+^ contents, and subsequently suppressed mammalian sirtuin activity [39,40,41]. In contrast, boosting NAD^+^ levels with biosynthetic precursors, such as nicotinamide riboside (NR), has been shown to stimulate sirtuin activity [42,43]. It becomes clear that sirtuins serve as “NAD^+^ sensors” to couple cellular energy status to their downstream signaling pathways. 

NAM, on the other hand, has been suggested as a pan-sirtuin inhibitor [44,45]. It depletes the imidate intermediate to favor the reverse “base exchange”, leading to inhibition of the forward deacetylation [36]. However, diverse NAM regulatory mechanisms have been observed for sirtuins. For mouse Sir2α and human SIRT2, complete inhibition can be achieved with increasing concentrations of NAM [36,46]. Human SIRT6 and *Archaeoglobus fulgidus* Sir2 (Af2Sir2), on the contrary, were only partially inhibited even at millimolar NAM concentrations [36,47]. Furthermore, the distinct activities of human SIRT5 exhibited different sensitivities to NAM inhibition: the desuccinylase activity was potently suppressed by NAM, while deacetylation was quite inert to NAM treatment [48]. The differential regulation of sirtuin activity by NAM warrants thorough mechanistic investigations.

Herein, we report detailed kinetic analysis of the SIRT1-catalyzed reactions using physiologically relevant peptide substrates. Robust deacetylation was observed for all the peptide substrates examined, consistent with a previous study [49]. Intriguingly, SIRT1-mediated base exchange demonstrated substrate preference. Histone peptides and p53-based peptide strongly supported incorporation of exogenously added radiolabeled NAM into NAD^+^ through base exchange. However, inefficient formation of radiolabeled NAD^+^ was obtained when p300-based peptide was used. The efficiency of base exchange vs. deacetylation has been suggested as a predictor of NAM inhibition [36]. Indeed, with the addition of millimolar concentrations of NAM, the deacetylation of histone peptides and p53 peptide were almost completely inhibited, and the deacetylation of p300 peptide was partially inhibited. Furthermore, the pH profiles and solvent isotope effects (SIEs) of the deacetylation and base exchange were also investigated. Deacetylation was pH-dependent with a normal SIE, indicative of a rate-limiting step involving proton transfer mediated by a titratable group. Base exchange, however, was largely pH-independent with no apparent SIE, consistent with the lack of proton transfer in the reaction mechanism. Collectively, our data suggested that NAM inhibition is governed by kinetic partitioning of the imidate intermediate, not by NAM binding *per se*.

## 2. Materials and Methods

### 2.1. Reagents and Instrument

All reagents were purchased from Aldrich (St. Louis, MO, USA) or Fisher Scientific (Pittsburgh, PA, USA) and were of the highest purity commercially available. UV spectra were obtained with a Varian Cary 300 Bio UV-visible spectrophotometer (Agilent Technologies, Santa Clara, CA, USA). HPLC was performed on a Dionex Ultimate 3000 HPLC system (Thermo Electron, Madison, WI, USA) equipped with a diode array detector using a Macherey-Nagel C18 reverse-phase column (Macherey-Nagel, Bethlehem, PA, USA). Radiolabeled samples were counted in a Beckman LS6500 scintillation counter (Beckman, Indianapolis, IN, USA).

### 2.2. Synthetic Peptides 

Synthetic peptides H3K9Ac: ARTKQTAR(K-Ac)STGGKAPRKQLAS, H4K16Ac: SGRGKGGKGLGKGGA(K-Ac)RHR, p300K1024Ac: ERSTELKTEI(K-Ac)EEEDQPSTS, p53K382Ac: KKGQSTSRHK(K-Ac)LMFKTEG were synthesized and purified by Genscript (Piscataway, NJ, USA). The peptides were purified by HPLC to a purity >95%.

### 2.3. Protein Expression and Purification 

Plasmid of SIRT1 (full length) was the generous gift from Dr. Hening Lin (Cornell University). The protein was expressed and purified according to a previously published protocol (Figure S1) [50]. The identity of the protein was confirmed by tryptic digestion followed by LC-MS/MS analysis performed at the Vermont Genetic Network (VGN) Proteomics Facility. Protein concentration was determined by Bradford assay.

### 2.4. Deacetylation Assay 

The *K*_m_ and *k*_cat_ of SIRT1 were measured for the synthetic peptide substrates. A typical reaction was performed in 100 mM phosphate buffer, pH 7.5, in a total volume of 50 μL. The reactions contained 500 μM NAD^+^ and increasing concentrations of peptide substrate. Reactions were initiated by the addition of 0.5 μM of SIRT1 and were incubated at 37 °C for 10 min before being quenched by 8 μL of 10% trifluoroacetic acid (TFA). The samples were then injected on an HPLC fitted to a Macherey-Nagel Nucleosil C18 column. The peaks of co-substrate NAD^+^ and products nicotinamide (NAM) and *O*-acetyl-ADP-ribose (AADPR) were resolved using a gradient of 0 to 20% methanol in 20 mM ammonium acetate. Chromatograms were analyzed at 260 nm. Reactions were quantified by integrating areas of peaks corresponding to NAD^+^ and the deacetylation product AADPR. Rates were plotted as a function of substrate concentration and the best fit of the points to the Michaelis–Menten equation was performed by Kaleidagraph^®^.

### 2.5. Nicotinamide Inhibition Assay 

To determine nicotinamide inhibition, reactions were performed in 100 mM phosphate buffer, pH 7.5, containing 500 μM NAD^+^, 500 μM peptide substrate, and various concentrations of NAM. The reactions were initiated by the addition of 0.5 μM of SIRT1 and were incubated at 37 °C for 30 min before being quenched by 8 μL of 10% TFA. The samples were then injected on an HPLC fitted to a Macherey-Nagel Nucleosil C18 column. Acetylated and deacetylated peptides were resolved using a gradient of 10% to 40% acetonitrile in 0.1% TFA. Chromatograms were analyzed at 215 nm. Reactions were quantified by integrating area of peaks corresponding to acetylated and deacetylated peptides. Rates were plotted as a function of NAM concentration, and points were fitted to Equation (1):

(1) $$\nu =\nu_{0}-\nu_{inh}\left(\frac{\left[I\right]}{K_{i}+\left[I\right]}\right)$$

where ν is the rate observed for a given concentration of NAM; ν_0_ is the uninhibited rate; ν_inh_ is the maximal inhibition; *K*_i_ is the apparent inhibition constant; and [*I*] is the concentration of NAM.

### 2.6. ^14^C-Nicotinamide Base Exchange Assay 

The reactions were carried out in 100 mM phosphate buffer, pH 7.5, containing 500 μM NAD^+^, 500 μM peptide substrate, 300,000 cpm [carbonyl-^14^C]-nicotinamide (^14^C-NAM, American Radiolabeled Chemicals Inc., St. Louis, MO, USA), and various concentrations of NAM. The reactions were initiated by the addition of 0.5 μM of SIRT1 and were incubated at 37 °C for 10 min before being quenched by 8 μL of 10% TFA. The samples were then injected on an HPLC fitted to a Macherey-Nagel Nucleosil C18 column. NAD^+^ and NAM were resolved using a gradient of 0 to 20% methanol in 0.1% TFA. Fractions containing NAM and NAD^+^ were collected and the radioactivity determined by scintillation counting. Rates were expressed as cpm/s incorporated into NAD^+^, and converted to turnover rate (s^−1^) after adjustment for specific radioactivity and enzyme concentration. Rates were plotted as a function of NAM concentration and the best fit of the points to the Michaelis–Menten equation was performed by Kaleidagraph^®^.

### 2.7. pH-Dependent Deacetylation Assay 

The pH effects on deacetylation was determined over a pH range of 6 to 9.5. Reactions contained 500 μM NAD^+^ and 500 μM p53K382Ac in a 100 mM phosphate buffer of pH differing across the full pH range by 0.25 pH units. The reactions were initiated by the addition of 0.5 μM SIRT1 and incubated at 37 °C for 10 min before being quenched by 8 μL of 10% TFA. Product formation was quantified by HPLC assay as described above in the “Deacetylation Assay” section. Rates were plotted as a function of pH, and points were fitted to Equation (2):

log *k*_cat_ = log *k* + log (*c*/(1+[H^+^]/*K*_a_)) (2)

where *k* is the rate at low pH; *k*_cat_ is the observed rate at a given pH; *c* is rate increment at high pH; and *K*_a_ is the active site ionization constant. 

### 2.8. pH-Dependent Base Exchange Assay 

The pH effects on base exchange was determined over a pH range of 6 to 9.5. Reactions contained 500 μM NAD^+^, 500 μM p53K382Ac and 2 mM ^14^C-NAM in a 100 mM phosphate buffer of pH differing across the full pH range by 0.25 pH units. The reactions were initiated by the addition of 0.5 μM SIRT1 and incubated at 37 °C for 10 min before being quenched by 8 μL of 10% TFA. Product formation was quantified by HPLC assay as described above in the “^14^C-Nicotinamide Base Exchange Assay” section. Rates were plotted as a function of pH.

### 2.9. Solvent Isotope Effects on Deacetylation 

Buffer components were added directly to D_2_O, and the pD values were determined by adding 0.4 to the reading of the pH electrode [51]. The pD effects on deacetylation was determined over a pD range of 6 to 9.5. Reactions contained 500 μM NAD^+^ and 500 μM p53K382Ac in a 100 mM phosphate buffer of pD differing across the full pD range by 0.25 pD units. The reactions were initiated by the addition of 0.5 μM SIRT1 and incubated at 37 °C for 10 min before being quenched by 8 μL of 10% TFA. Product formation was quantified by HPLC assay as described above in the “Deacetylation Assay” section. The rates were plotted as a function of pD. The solvent isotope effects (SIEs) were expressed as SIE = *k*_H2O_/*k*_D2O_. 

### 2.10. Solvent Isotope Effects on Base Exchange 

The SIE effect on base exchange was determined at pH (pD) 8.5. The reactions were carried out in a 100 mM phosphate buffer containing 500 μM NAD^+^, 500 μM peptide substrate, 300,000 cpm ^14^C-NAM, and various concentrations of NAM. The reactions were initiated by the addition of 0.5 μM of SIRT1 and were incubated at 37 °C for 10 min before being quenched by 8 μL of 10% TFA. Product formation was quantified by HPLC assay as described above in the “^14^C-Nicotinamide Base Exchange Assay” section. Rates were plotted as a function of pH (pD). The SIE was expressed as SIE (*k*_cat_/*K*_m_) = (*k*_cat_/*K*_m_)^H2O^/(*k*_cat_/*K*_m_)^D2O^. 

## 3. Results and Discussions

### 3.1. SIRT1 Catalyzes Substrate-Dependent Base Exchange 

**Figure 1:**
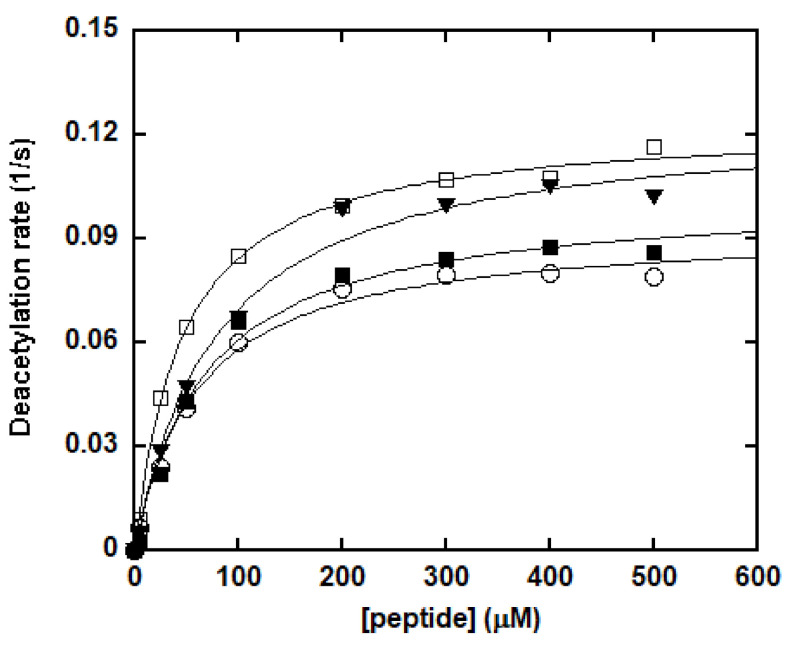
Steady-state kinetic analysis of SIRT1-catalyzed deacetylation. Peptide saturation curves for p53K382Ac (closed square), H3K9Ac (triangle), H4K16Ac (open square) and p300K1024Ac (circle). Reactions containing 500 μM NAD^+^, and various concentrations of peptide substrate in a 100 mM phosphate buffer, pH 7.5, were initiated by the addition of 0.5 μM of SIRT1. The reactions were incubated at 37 °C for 10 min and quenched by 8 μL of 10% TFA. Rates were determined as described in the “Materials and Methods” section. Rates were plotted as a function of peptide concentration and fit to the Michaelis–Menten equation using Kaleidagraph^®^.

Among the seven human sirtuins, SIRT1 is the most studied isoform with a wealth of cellular targets [52]. Our initial effort focused on the biochemical characterization of recombinant SIRT1 activity using synthetic peptides derived from endogenous SIRT1 substrates including histones H3 and H4, tumor suppressor p53, and transcription coactivator p300. Table 1 lists the steady-state kinetic parameters for the SIRT1-catalyzed reactions. The deacetylation activity was evaluated using an HPLC-based assay as described in the “Materials and Methods” section. Full-length recombinant human SIRT1 was able to deacetylate all the peptide substrates examined in an NAD^+^-dependent fashion (Figure 1). The *K*_m_ values were determined to be 68.5 ± 9.7 μM for p53K328Ac, 79.6 ± 10.8 μM for H3K9Ac, 46.4 ± 2.9 μM for H4K16Ac and 60.9 ± 6.7 μM for p300K1024Ac, respectively. The *k*_cat_ values were in the range from 0.093 ± 0.003 to 0.125 ± 0.005 s^−1^. H4K16Ac demonstrated the highest catalytic efficiency of 2.67 × 10^−3^ s^−1^M^−1^, 1.7 times higher than that of p53K382Ac, stemming primarily from the high binding affinity. Overall, the catalytic efficiency of the deacetylation was comparable for this panel of synthetic peptides. 

**Figure 2:**
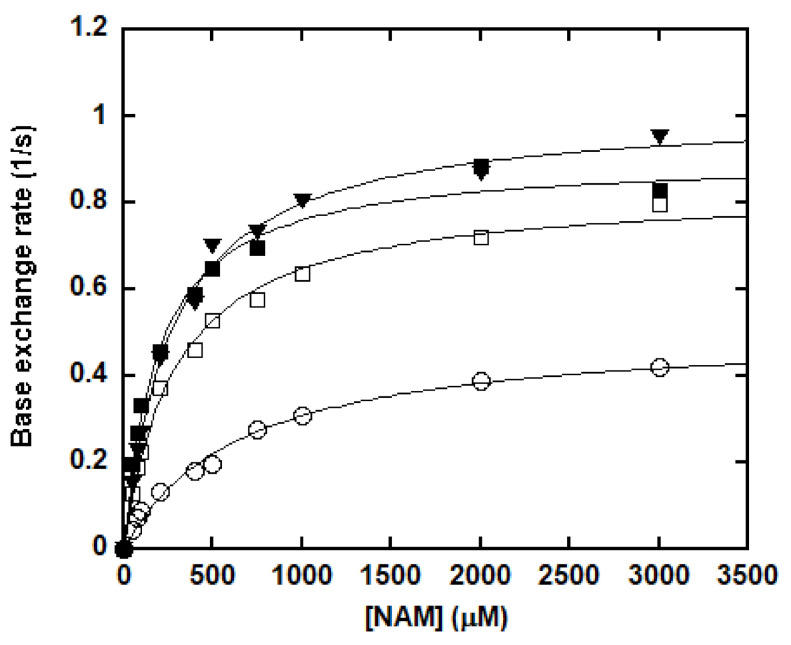
Kinetics of the SIRT1-catalyzed base exchange of ^14^C-NAM into unlabeled NAD^+^. The reactions were carried out in a 100 mM phosphate buffer, pH 7.5, containing 500 μM NAD^+^, 500 μM peptide substrate (p53K382Ac: closed square; H3K9Ac: triangle; H4K16Ac: open square; and p300K1024Ac: circle), 300,000 cpm ^14^C-NAM, and various concentrations of NAM. The reactions were initiated by the addition of 0.5 μM of SIRT1 and incubated at 37 °C for 10 min before being quenched by 8 μL of 10% TFA. Rates were determined as described in the “Materials and Methods” section and were plotted as a function of NAM concentration. The points were fitted to the Michaelis–Menten equation using Kaleidagraph^®^.

The base exchange reactions were performed with SIRT1, saturating concentrations of NAD^+^ and peptide substrate, 3 × 10^5^ cpm [carbonyl-^14^C]-nicotinamide (^14^C-NAM) as well as increasing concentrations of NAM. NAD^+^ and NAM were then resolved by HPLC, collected separately, and subjected to scintillation counting. Counts per minute (cpm) values were converted to the concentrations of ^14^C-NAD^+^ for the calculation of the kinetic parameters. Surprisingly, SIRT1-catalyzed base exchange showed substrate preference (Figure 2 and Table 1). P300K1024Ac had the lowest catalytic efficiency of 0.78 × 10^−3^ s^−1^M^−1^ with high *K*_m_ (652.5 ± 71.6 μM) and low *k*_cat_ (0.509 ± 0.022 s^−1^). The catalytic efficiency of H3K9Ac and H4K16Ac were 3.74 × 10^−3^ and 2.93 × 10^−3^ s^−1^M^−1^, respectively. The nearly 4.8-fold and 3.8-fold increases compared to p300K1024Ac can be attributed to improved *K*_m_ and *k*_cat_. More strikingly, p53K382Ac exhibited a nearly 6-fold increase in catalytic efficiency in comparison with p300K1024Ac, owing to a 3.4-fold reduction of *K*_m_ (191.0 ± 16.2 μM) and a 1.8-fold increase of *k*_cat_ (0.905 ± 0.020 s^−1^). Previous characterizations of SIRT1 have been focusing on its deacetylase activity, which exhibits certain sequence selectivity [53,54]. Our study was the first one to illustrate the substrate-dependent base exchange activity of SIRT1, consistent with the partitioning behavior of the imidate intermediate (Scheme 1).

### 3.2. Peptide Substrates Demonstrate Differential Sensitivity to NAM Inhibition 

**Figure 3:**
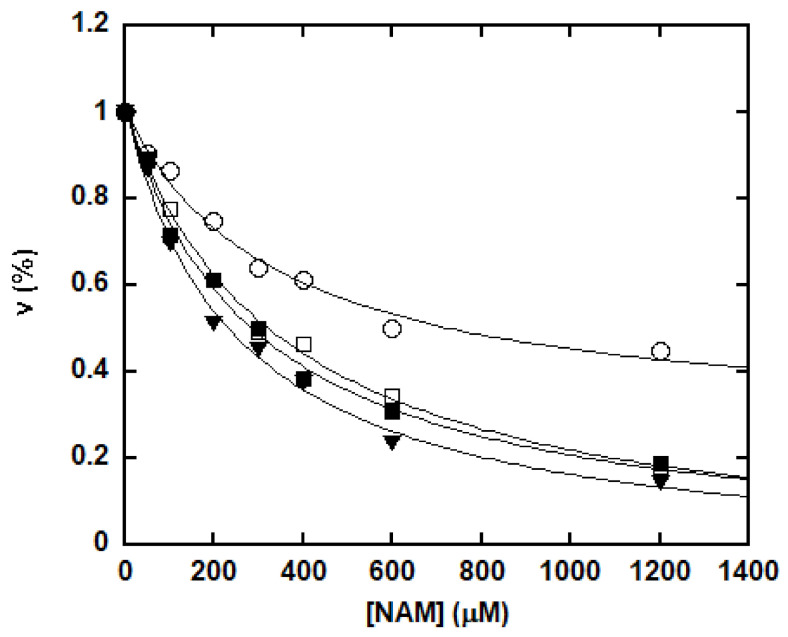
Inhibition of the deacetylation by increasing concentrations of NAM. The reactions were performed in a 100 mM phosphate buffer, pH 7.5, containing 500 μM NAD^+^, 500 μM peptide substrate (p53K382Ac: closed square; H3K9Ac: triangle; H4K16Ac: open square; and p300K1024Ac: circle) and various concentrations of NAM. The reactions were initiated by the addition of 0.5 μM of SIRT1 and were incubated at 37 °C for 30 min before being quenched by 8 μL of 10% TFA. Rates were determined as described in the “Materials and Methods” section and were plotted as a function of NAM concentration. The points were fitted to the equation ν = ν_0_ − ν_inh_ $\left(\frac{\left[I\right]}{K_{i}+\left[I\right]}\right)$ using Kaleidagraph^®^.

NAM is not only a byproduct, but also an inhibitor of sirtuin-catalyzed deacetylation [36,46]. NAM inhibition was assayed with saturating concentrations of NAD^+^ and peptide, together with increasing concentrations of NAM as described in the “Materials and Methods” section. The percentage of remaining rate of deacetylation was plotted as a function of NAM concentration (Figure 3). Decreasing deacetylation was observed for all the peptide substrates with increasing levels of NAM. For p53K382Ac, H3K9Ac and H4K16Ac, greater than 80% inhibition occurred at high NAM concentration. However, NAM did not cause complete inhibition for p300K1024Ac. For this peptide, an approximately 55% reduction of the deacetylation rate was obtained at millimolar NAM concentrations.

**Scheme 2:**
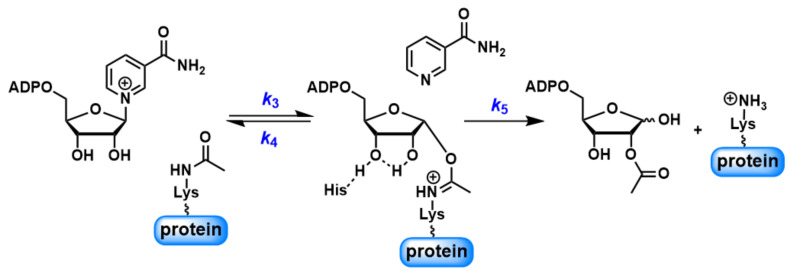
Abbreviated scheme for the SIRT1-catalyzed reactions.

It has been suggested that forward deacetylation and reverse base exchange compete for the same imidate intermediate (Scheme 2) [36,46]. In other words, the rate of return to the Michaelis complex (*k*_4_) competes with the forward rate of nucleophilic attack of 2′-OH to the imidate (*k*_5_). If *k*_4_ > *k*_5_, when sufficient NAM is available either at physiological NAM concentrations or near the *K*_i(NAM)_ of the enzyme, a significant fraction of imidate cannot proceed forward efficiently, leading to inhibition of the deacetylation. The *k*_cat_ of deacetylation for SIRT1 was ~0.1 s^−1^. All four peptides showed rapid base exchange rates in the range of 0.509 to 1.015 s^−1^, suggesting a kinetically competent reverse process. The *K*_i(NAM)_ values for p53K382Ac, H3K9Ac and H4K16Ac were 294.4 ± 44.6, 247.9 ± 35.1 and 356.6 ± 38.2 μM, respectively (Table 1), in good agreement with their *K*_m_ values for base exchange. P300K1024Ac exhibited the lowest *k*_cat(exchange)_/*k*_cat(deacetylation)_ of 5.5, consistent with the partial inhibition observed at saturating NAM concentrations.

### 3.3. SIRT1 Demonstrates pH-Dependent Deacetylation and pH-Independent Base Exchange 

**Figure 4:**
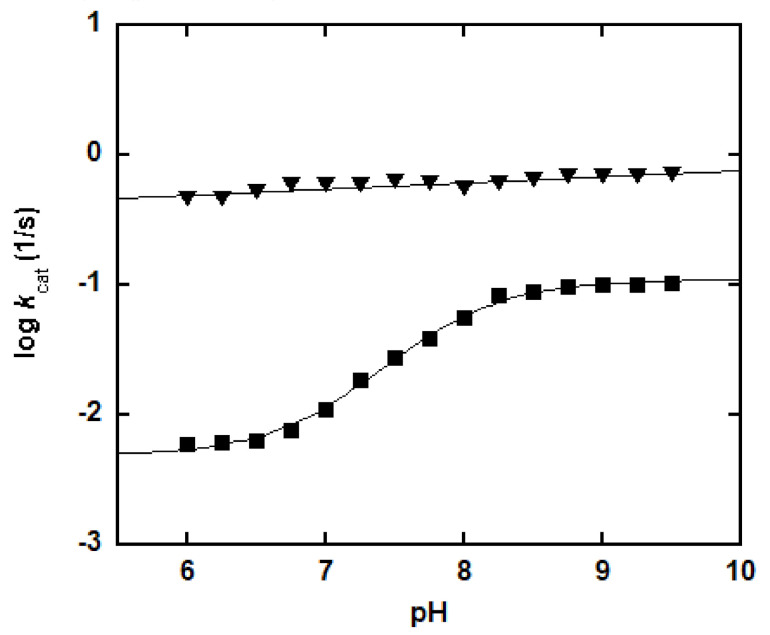
pH dependence of the SIRT1-catalyzed reactions. pH effects on the SIRT1-catalyzed deacetylation (closed square) and base exchange (triangle) were determined over a pH range of 6 to 9.5. The deacetylation reactions contained 500 μM NAD^+^ and 500 μM p53K382Ac in a 100 mM phosphate buffer of pH differing across the full pH range by 0.25 pH units. The reactions were initiated by the addition of 0.5 μM SIRT1 and incubated at 37 °C for 10 min before being quenched by 8 μL of 10% TFA. Rates were determined as described in the “Materials and Methods” section and were plotted as a function of pH. The points were fitted to the equation log *k*_cat_ = log *k* + log (*c*/(1 + [H^+^]/*K*_a_)) using Kaleidagraph^®^. The p*K*_a_ was determined to be 7.37. The base exchange reactions contained 500 μM NAD^+^, 500 μM p53K382Ac and 2 mM ^14^C-NAM in a 100 mM phosphate buffer of pH differing across the full pH range by 0.25 pH units. The reactions were initiated by the addition of 0.5 μM SIRT1 and incubated at 37 °C for 10 min before being quenched by 8 μL of 10% TFA. Rates were determined as described in the “Materials and Methods” section and were plotted as a function of pH. The points were fitted to a linear line.

To further probe the partitioning of the imidate intermediate, the pH-dependence of both the deacetylation and base exchange was examined over a pH range of 6 to 9.5 using p53K382Ac as the substrate (Figure 4). Deacetylation was performed with saturating concentrations of NAD^+^ and peptide in buffer solutions of pH differing across the full pH range by 0.25 pH units. The deacetylation rate was largely unchanged between pH 6 and 7, and then increased in magnitude in a manner consistent with change in the protonation state of a titratable residue. The deacetylation rate increased approximately 14-fold at pH 8.25 and reached a plateau beyond this pH. The pH profile of the deacetylation defined a critical ionization with a p*K*_a_ value of 7.37, consistent with the p*K*_a_ of an active site histidine (H363 for SIRT1), which was proposed to act as a general base to activate 2′-OH (Scheme 2) [55]. These results strongly support the notion that *k*_5_, but not product release, is the rate-limiting step for deacetylation.

In contrast, SIRT1-mediated base exchange was insensitive to pH changes (Figure 4). The rate of base exchange gradually increased with pH, showing merely a 1.7-fold increase from pH 6 to 9.5. The data were obtained with saturating concentrations of NAD^+^ and peptide in combination of 2 mM ^14^C-NAM. The steady-state kinetics of base exchange over a range of NAM concentrations was further evaluated at pH 6.5 and 8.5 to confirm the pH-independency (Figure S2). The *K*_m_ values remained roughly unchanged at different pH values (118.6 ± 11.1 μM at pH 6.5, and 126.0 ± 10.1 μM at pH 8.5, respectively). The *k*_cat_ values increased from 0.616 ± 0.016 s^−1^ at pH 6.5 to 0.684 ± 0.016 s^−1^ at pH 8.5, consistent with the absence of proton transfer in the reverse process.

The pH-dependence analysis revealed that *k*_5_ increased with pH, while *k*_4_ remained nearly constant across the entire pH range examined (Scheme 2). Since imidate partitioning governs NAM inhibition, it was proposed that an increasing pH would weaken this inhibition. The NAM inhibition assays were performed at pH 6.5 and 8.5 (Figure S3). With increasing concentrations of NAM, the residue deacetylation rate decreased at both pH values. However, the maximal inhibition at 1.2 mM NAM declined from nearly 90% at pH 6.5 to 68% at pH 8.5, implying that elevating pH could serve as an effective strategy to relieve NAM inhibition.

### 3.4. SIRT1-Catalyzed Deacetylation Exhibits Normal Solvent Isotope Effects (SIEs) 

**Figure 5:**
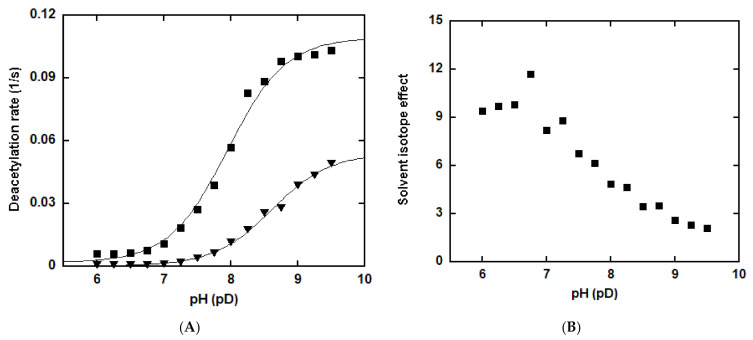
Solvent isotope effects of SIRT1-catalyzed deacetylation. (**A**) pH (pD) effects on deacetylation was determined over a pH (pD) range of 6 to 9.5. Reactions contained 500 μM NAD^+^ and 500 μM p53K382Ac in a 100 mM phosphate buffer of pH (pD) differing across the full pH (pD) range by 0.25 pH (pD) units. The reactions were initiated by the addition of 0.5 μM SIRT1 and incubated at 37 °C for 10 min before being quenched by 8 μL of 10% TFA. Rates were determined as described in the “Materials and Methods” section and plotted as a function of pH (pD). Closed square: in water; Triangle: in D_2_O; (**B**) SIEs on deacetylation. The SIEs were expressed as SIE = *k*_H2O_/*k*_D2O_ and plotted as a function of pH (pD).

SIEs are informative in determining the involvement of proton transfer in the rate-limiting step [51]. They can be expressed as a function of pH (pD) because of kinetically essential ionizations of exchangeable positions in either the enzyme or the substrate. The rate of deacetylation using p53K382Ac as the substrate was measured in H_2_O and D_2_O at various pH (pD) values between 6 and 9.5. Figure 5A shows the plots of reaction rate as a function of pH (pD). Activity increased with the pH (pD) in either the proteated or deuterated buffers, and appeared to be lower in D_2_O. Normal SIEs were detected at all the pH (pD) values and gradually declined from 9.4 at pH (pD) 6 to 2.1 at pH (pD) 9.5 (Figure 5B). Transfer of solvent-sensitive protons yields SIEs along the reaction coordinate. The results provided direct evidence of a kinetically significant ionization during the reaction, consistent with *k*_5_ being rate-limiting since proton transfer mediated by H363 is inherent of the step (Scheme 2).

**Figure 6:**
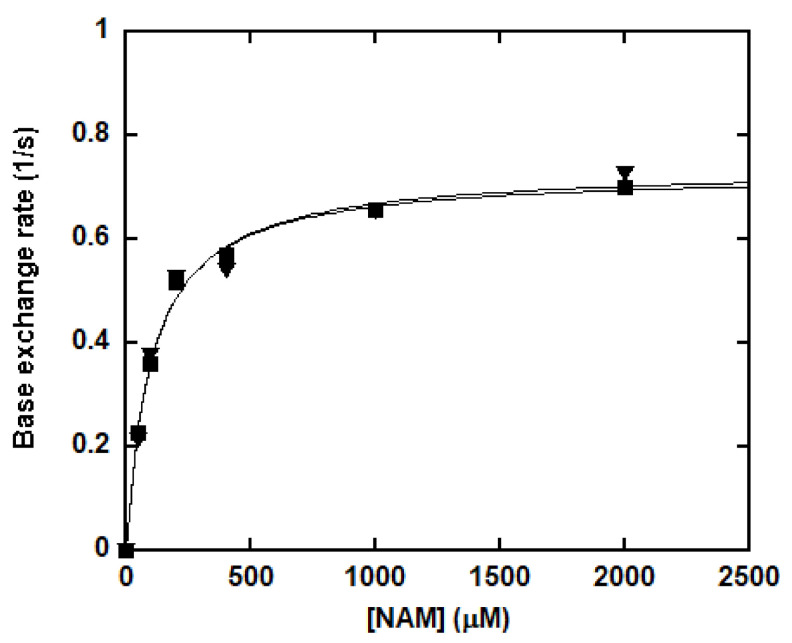
Solvent isotope effects (SIE) of SIRT1-catalyzed base exchange. The SIEs effect on base exchange was determined at pH (pD) 8.5. The reactions were carried out in a 100 mM phosphate buffer containing 500 μM NAD^+^, 500 μM peptide substrate, 300,000 cpm ^14^C-NAM and various concentrations of NAM. The reactions were initiated by the addition of 0.5 μM of SIRT1 and were incubated at 37 °C for 10 min before being quenched by 8 μL of 10% TFA. Rates were determined as described in the “Materials and Methods” section and plotted as a function of NAM concentrations. Closed square: in water; Triangle: in D_2_O.

Our previous study indicated that SIRT1-catalyzed base exchange was pH-independent. No SIE was expected for this reverse process. The SIE on *k*_cat_/*K*_m_ for base exchange at pH (pD) 8.5 was examined as described in the “Materials and Methods” section (Figure 6). The *k*_cat_ and *K*_m_ at pH 8.5 were determined to be 0.730 ± 0.014 s^−1^ and 100.4 ± 7.9 μM, respectively. The *k*_cat_ and *K*_m_ at pD 8.5 were 0.739 ± 0.029 s^−1^ and 104.7 ± 16.6 μM, respectively. The SIE (*k*_cat_/*K*_m_) was calculated to be 1.03 ± 0.06, in accord with proton transfernot being a kinetically significant step in base exchange. The absence of an SIE suggested that all the chemical steps are relatively fast compared to the steady-state rate of turnover. 

**Figure 7:**
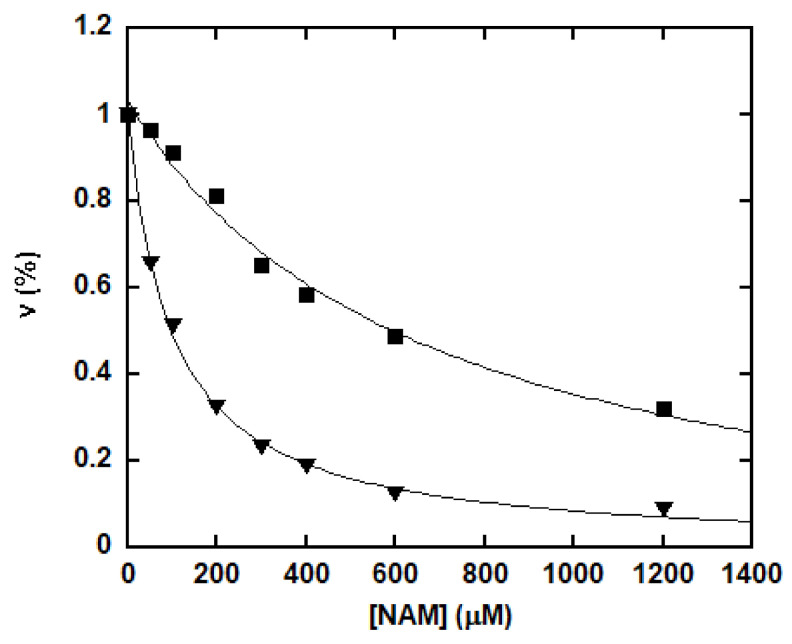
Solvent isotope effects of NAM inhibition. The reactions were performed in a 100 mM phosphate buffer at pH 8.5 (closed square) or pD 8.5 (triangle) containing 500 μM NAD^+^, 500 μM p53K382Ac and various concentrations of NAM. The reactions were initiated by the addition of 0.5 μM of SIRT1 and were incubated at 37 °C for 30 min before being quenched by 8 μL of 10% TFA. Rates were determined as described in the “Materials and Methods” section and plotted as a function of NAM concentration. The points were fitted to the equation ν = ν_0_ − ν_inh_$\left(\frac{\left[I\right]}{K_{i}+\left[I\right]}\right)$ using Kaleidagraph^®^.

The significant SIEs for deacetylation and the lack of SIEs for base exchange prompted us to investigate the SIEs on NAM inhibition of the forward deacetylation, which is inversely proportional to base exchange. NAM inhibition curves were obtained at pH 8.5 and pD 8.5 (Figure 7). The maximal inhibition improved from 68% at pH 8.5 to greater than 90% at pD 8.5, indicating NAM is a better inhibitor for the deacetylation reaction in D_2_O.

## 4. Conclusions and Perspectives

SIRT1-catalyzed reactions couple NAD^+^ and acetyllysine substrate to form an imidate intermediate. Subsequently, the imidate can either move forward for deacetylation or return backwards to reform NAD^+^ through the “base exchange” process (Scheme 2). The imidate intermediate is long-lived to equilibrate NAM and serves as a “checkpoint” in the reaction mechanism [56]. The study of SIRT1 biochemical activity has been quite controversial. An early study suggested no substrate specificity for SIRT1 in vitro [49]. However, recent evidence indicated SIRT1 recognizes its substrate in a sequence-dependent manner [53,54]. It is important to point out that these previous studies have been focusing on the deacetylation activity. The substrate selectivity of base exchange has not been explored. In the current study, SIRT1-catalyzed reactions were investigated using a panel of synthetic peptide substrates based on several physiological SIRT1 targets. As one of the human sirtuins with robust deacetylase activity, SIRT1 was able to deacetylate both histone and non-histone peptides with high efficiency. Kinetically competent reverse base exchange reactions were also observed for all the peptide examined, with *k*_cat(exchange)_/*k*_cat(deacetylation)_ in the range of 5.5 to 8.8. Particularly, p300K1024Ac exhibited the lowest catalytic efficiency of base exchange, approximately 6 times lower than that of p53K382Ac. Inefficient base exchange implied resistance or insensitivity to NAM inhibition. Indeed, the deacetylation of p300K1024Ac cannot be completely inhibited at physiological NAM concentrations, in sharp contrast to the behaviors of other peptides.

In the deacetylation mechanism, a highly conserved active site histidine residue has been proposed to act as a general base to deprotonate and activate the 2′-OH of the ribose moiety for the subsequent nucleophilic attack of the imidate intermediate [55,57,58]. Our pH-dependence study supported this hypothesis, as evidenced by the increased deacetylation rate with increasing pH, which centered at pH of 7.37, consistent with the p*K*_a_ of the active site H363. Conversely, SIRT1-mediated base exchange was largely pH-independent, indicating no significant participation of H363 in the reverse process. The decreased *k*_5_/*k*_4_ as a function of pH suggested that NAM inhibition can be weakened at elevated pH. Indeed, the maximal inhibition at saturating NAM concentration was significantly reduced by going from pH 6.5 to pH 8.5. 

To investigate the proton transfer step, SIEs were also measured. Normal SIEs were detected for deacetylation, further confirming that proton transfer is involved in the rate-limiting step for the forward reaction. The SIE measured for base exchange at pH (pD) 8.5 was close to unity, indicative of proton transfer not being a kinetically significant step for the reverse process. Furthermore, NAM inhibition was evaluated at pH (pD) 8.5. The inhibition was strengthened in the deuterated solvent, resulting in a 22% increase in maximal inhibition at millimolar NAM concentration. 

“Activation by de-repression” has been proposed as an effective sirtuin activation strategy, as evidenced by the development of isonicotinamide as a sirtuin activator [37]. Alternatively, depletion of NAM by nicotinamidase, an enzyme responsible for converting NAM to nicotinic acid (NA), has been shown to activate Sir2 [59,60]. Our results suggested that NAM inhibition can also be relieved at elevated pH in proteated solvent, owing to the pH-sensitive deacetylation and pH-insensitive base exchange. pH engineering in enzyme microenvironment has been shown to enhance enzymatic activity [61,62]. For example, covalent conjugation of cytochrome C (cyt C), an enzyme with an optimal pH of ~4.5, to highly negatively charged poly(methacrylic acid), created an acidic microenvironment near the enzyme active site. This modification led to a more than 2-fold activity increase compared to the unmodified enzyme [61]. Manipulating microenvironmental pH values also has significant therapeutic consequences. Alteration of tumor microenvironment pH values using small molecules such as bicarbonates and proton pump inhibitors offered a potential strategy to improve the efficacy of cancer therapy [63]. Our study not only revealed novel regulatory mechanism for mammalian sirtuins, but also opened new avenue of manipulating the pH profile to activate these enzymes.

## Figures and Tables

**Table 1:** Recombinant SIRT1 steady-state parameters.

Substrate	Deacetylation				Base Exchange		
	*K*_m_ (µM)	*k*_cat_ (s^−1^)	*k*_cat_/*K*_m_ (10^−3^s^−1^M^−1^)	*K*_i (NAM)_ (µM)	*K*_m_ (mM)	*k*_cat_ (s^−1^)	*k*_cat_/*K*_m_ (10^−3^s^−1^M^−1^)
**p53K382Ac**	68.5 ± 9.7	0.103 ± 0.004	1.50	294.4 ± 44.6	191.0 ± 16.2	0.905 ± 0.020	4.74
**H3K9Ac**	79.6 ± 10.8	0.125 ± 0.005	1.57	247.9 ± 35.1	271.5 ± 18.8	1.015 ± 0.021	3.74
**H4K16Ac**	46.4 ± 2.9	0.124 ± 0.002	2.67	356.6 ± 38.2	284.2 ± 22.1	0.832 ± 0.019	2.93
**p300K1024Ac**	60.9 ± 6.7	0.093 ± 0.003	1.53	336.1 ± 68.7	652.5 ± 71.6	0.509 ± 0.022	0.78

## Data Availability

The data presented in this study are available in the main text of this article and the supplementary material.

## Supplementary Materials

The following are available online at https://www.mdpi.com/2218-273X/11/2/312/s1, Figure S1: SDS-PAGE image of purified recombinant human SIRT1; Figure S2: SIRT1-catalyzed pH-independent base exchange; Figure S3: pH effects on NAM inhibition.
