# Supplementary material for: Substrate-Dependent Sensitivity of SIRT1 to Nicotinamide Inhibition
Source: Biomolecules. 2021 Feb 18;11(2):312. doi: 10.3390/biom11020312 (PMC7922766; doi:10.3390/biom11020312)
Supplement: Supplementary file 1 [file biomolecules-11-00312-s001.pdf]

Supporting Information

Substrate-dependent Sensitivity of SIRT1 to Nicotinamide Inhibition

Stacia Rymarchyk,[†] Wenjia Kang,[‡] Yana Cen[‡,#,*]

[†]*Department of Pharmaceutical Sciences, Albany College of Pharmacy and Health Sciences, Colchester, VT 05446*

[‡]*Department of Medicinal Chemistry, Virginia Commonwealth University, Richmond, VA 23219*

[#]*Institute for Structural Biology, Drug Discovery and Development, Virginia Commonwealth University, Richmond, VA 23219*

[*]Correspondence: ceny2@vcu.edu, phone: 804-828-7405

Figure S1. SDS-PAGE image of purified recombinant human SIRT1.

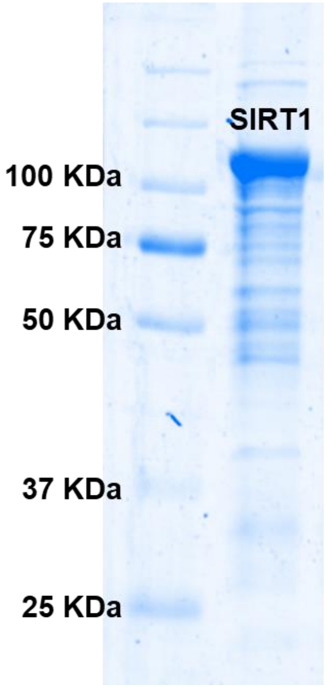

Figure S2. SIRT1-catalyzed pH-independent base exchange.

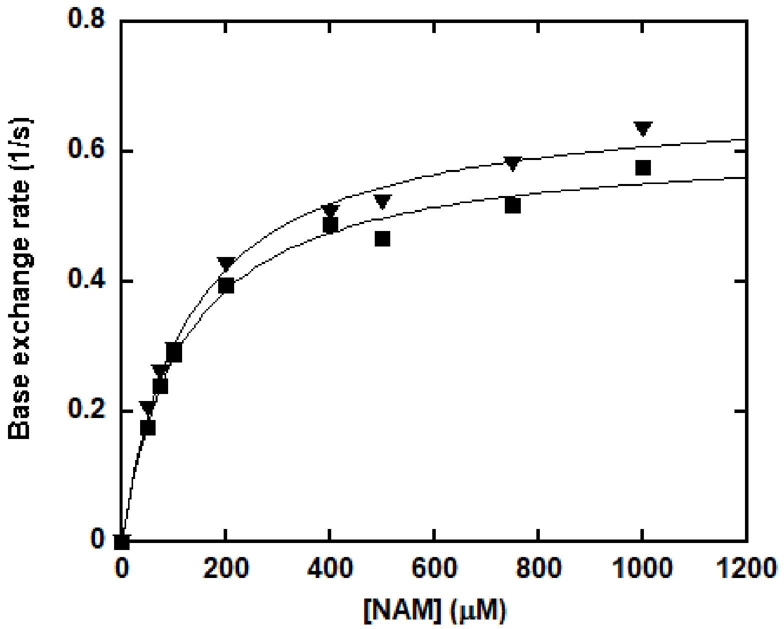

Base exchange reactions were performed at pH 6.5 (closed square) and 8.5 (triangle). The reactions were carried out in 100 mM phosphate buffer containing 500 μM NAD^+, 500 μM p53K382Ac, 300,000 cpm ^{14}C-NAM, and various concentrations of NAM. The reactions were initiated by the addition of 0.5 μM of SIRT1 and were incubated at 37°C for 10 min before being quenched by 8 μL of 10% TFA. Rates were determined as described in "Materials and Methods", plotted as a function of NAM concentration, and best fits of points to the Michaelis-Menten equation were performed by Kaleidagraph®.

Figure S3. pH effects on NAM inhibition.

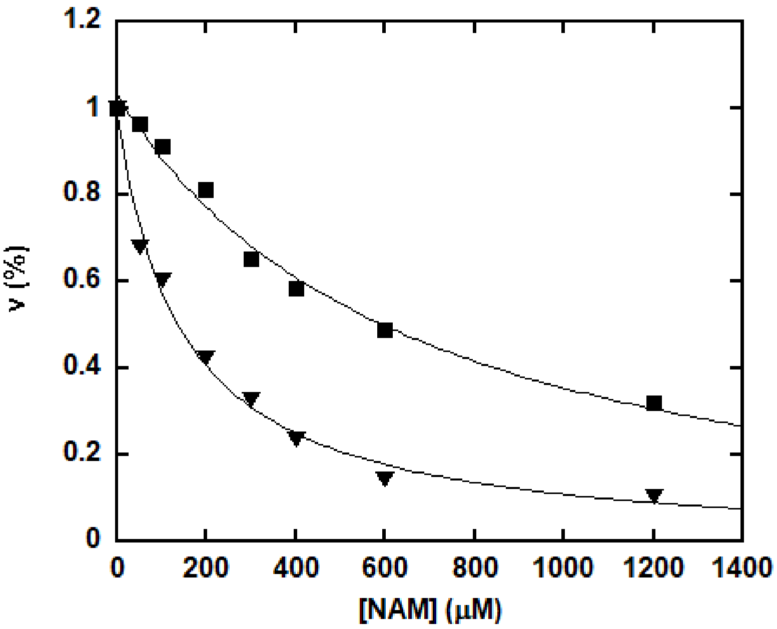

The reactions were performed in 100 mM phosphate buffer at pH 6.5 (triangle) or 8.5 (closed square) containing 500 μM NAD^+, 500 μM p53K382Ac, and various concentrations of NAM. The reactions were initiated by the addition of 0.5 μM of SIRT1 and were incubated at 37°C for 30 min before being quenched by 8 μL of 10% TFA. Rates were determined as described in “Materials and Methods” and plotted as a function of NAM concentration. The points were fitted to the equation: $\nu = \nu_0 - \nu_{inh} \left(\frac{[I]}{K_i + [I]}\right)$ using Kaleidagraph®.
